# Empirical evidence of the continuing improvement in cost efficiency of an endoscopic surveillance programme for gastric cancer in Singapore from 2004 to 2010

**DOI:** 10.1186/1472-6963-13-139

**Published:** 2013-04-15

**Authors:** Hui Jun Zhou, Shu Chuen Li, Nasheen Naidoo, Feng Zhu, Khay Guan Yeoh

**Affiliations:** 1 Saw Swee Hock School of Public Health, National University of Singapore, MD3, 16 Medical Drive, Singapore, Singapore; 2 Discipline of Pharmacy & Experimental Pharmacology, School of Biomedical Sciences & Pharmacy, University of Newcastle, Room 607c, Medical Sciences Building, Callaghan, NSW, 2308, Australia; 3 Department of Medicine, Yong Loo Lin School of Medicine, National University of Singapore, 1E, Kent Ridge Road, NUHS Tower Block Level 10, Singapore, Singapore

**Keywords:** Gastric cancer, Cancer prevention, Programme evaluation, Cost efficiency, Endoscopy, Direct cost, Generalized estimation equation

## Abstract

**Background:**

Endoscopic surveillance has been proven effective in prolonging the survival of gastric cancer (GC) patients. However, there is limited evidence on the cost efficiency of delivering this intervention, especially on a national level in spite of cost efficiency being a major determinant of the actual cost-effectiveness of a cancer prevention programme. The Singapore Gastric Cancer Epidemiology Clinical and Genetic Programme (GCEP) is a demonstration project offering scheduled endoscopy to the Chinese population aged 50 years or older in Singapore. By assessing the cost efficiency of the GCEP, this study aimed to provide empirical evidence on the cost structure and mechanisms underlying cost generation in conducting GC surveillance, thus informing resource allocation and programme budgeting for the Singapore government.

**Methods:**

From a societal perspective, we reported on the direct cost (resource consumption) of conducting endoscopic surveillance through the GCEP network. We retrospectively collected individual-level data of 216 subjects recruited at the National University Hospital, Singapore from 01/04/2004 to 31/10/2010. The Overall Cost, Clinical Cost, GCEP Cost and Personal Cost incurred in serving one subject was computed and discounted as 2004 US dollar (US$) per capita for every year. The Generalized Estimation Equation (GEE) was used to model the data.

**Results:**

All cost indices continuously declined over the 6.5-year costing period. For the total sample, Overall Cost, Clinical Cost, GCEP Cost and Personal Cost declined by 42.3%, 54.1%, 30% and 25.7% respectively. This downward trend existed for age and gender subgroups and the high risk group only with cost reductions varying between 3.5% and 58.4%. The GEE models confirmed statistical significance of the downward trend and of its association with risk profile, where the moderate risk group had cost indices at most 77% of the high risk group.

**Conclusions:**

Our study offered empirical evidence of improved cost efficiency of a surveillance programme for GC in the early phase of programme implementation. Mechanisms such as economies of scale and self-learning were found to be involved in the cost reduction. Our findings highlighted the importance of assessing the cost efficiency and offered valuable insights for future programme budgeting and policy making.

## Background

In the global campaign to eradicate gastric cancer (GC), which claims over 700,000 lives every year [1], screening has assumed a paramount role but not without limitations [2]. Even in Japan, the country with the highest incidence of GC, the cost-effectiveness of national GC screening is fading away due to the decrease in incident cases [3]. Considering the worldwide declining trend of GC incidence over the past two decades [4], screening at the national level may not be the optimal strategy for GC eradication. Therefore, GC surveillance targeted at high risk subpopulations offers a complementary or alternative strategy given that hospital-based GC surveillance has already demonstrated its efficacy in detecting cases at early stages of cancer development [5,6]. Surveillance is also sensible for industrialized countries with overall very low GC incidence because of specific ethnic groups such as Asian immigrants among whom GC remains a major disease burden [7,8].

The Gastric Cancer Epidemiology Clinical and Genetic Programme (GCEP) is an endoscopic surveillance programme targeted at the Chinese population aged 50 years or above in Singapore, a country with an intermediate risk of GC [9]. The GCEP target population has a GC incidence much higher than the general population [10]. Based on the preliminary explorations of GC endoscopic screening [11], the GCEP was intended to inform the feasibility and benefit of GC surveillance as a control strategy for GC in Singapore. The GCEP system is established in four local general hospitals and has been running since 2004. Its surveillance follow-up is incorporated into the daily work routine of the participating hospitals.

Cancer prevention programmes are costly undertakings and are featured by many years or even decades of time-lag between investment and the desired outcome. This then raises an important issue of cost efficiency - whether the programme is producing the service at the least cost, i.e., the lowest price per unit of service. Cost efficiency is one of major determinants of actual cost-effectiveness delivered by a specific programme [12]. However, to date, most cost analyses on cancer prevention have been cross-sectional studies which may have lead to skewed or biased cost estimates [13-15]. A survey-based top-down approach for data collection adopted in these studies is also prone to subjectivity [16]. The time-lag effect and long-term follow up associated with cancer prevention entails the understanding of continuous cost generation which a cross-sectional study is unable to address. Furthermore previous studies have not investigated the clinical cost, the patient personal cost and the programme associated cost in the same study, and are thus limited in providing a more detailed and complete description of the broader economic impact of the programme [17-21].

With the above considerations, a trial programme such as the GCEP is the ideal vehicle to empirically explore and evaluate the cost efficiency of GC surveillance. Evaluating the GCEP would be very informative for a future cost-effectiveness analysis of GC surveillance in Singapore and would have direct relevance to government budgeting. This will prevent incomplete information from affecting optimal resource allocation decisions. Thus, we designed this study with the aims of 1) informing resource allocation and programme budgeting in the planning of national surveillance of GC in Singapore; 2) providing a comprehensive cost structure for full economic evaluation of GC surveillance both locally and worldwide; 3) elucidating the mechanisms underlying cost generation of cancer surveillance programmes. This information will be of value to health administrators and planners in planning similar programmes, as well as providing a framework for health policy researchers to undertake similar studies in different jurisdictions.

## Methods

### General approach

The GCEP is an ongoing trial surveillance programme with the aim of eventually becoming a government-organized GC surveillance programme. The most pertinent concern about establishing such a programme is the financial impact on society by the programme. Therefore, this study was conducted from a societal perspective, whereby the direct costs on GCEP (the healthcare provider) and the patients (the beneficiaries) in undertaking GCEP surveillance were measured by a bottom-up approach and reported as US dollars per person served [22].

### Service mix of the GCEP programme

According to the GCEP Protocol Version 7, once a subject was recruited, the 5-year annual follow-up would be customized to the individual’s risk of developing GC assessed at baseline. Those fulfilling any of five criteria, namely (1) dysplasia, (2) intestinal metaplasia, (3) atrophic gastritis, (4) GC family history, and (5) presence of *H. pylori* infection were categorized as high risk subjects and underwent annual oesophago-gastro-duodenoscopy (OGD) examination. All other subjects were classified as moderate risk and underwent OGD in Years 3 and 5 with telephone interviews or clinic visits in Years 1, 2, and 4. If patients were unable to be present for endoscopy, they were contacted by telephone for GC related symptoms. The primary outcome was the detection of early gastric cancer or high grade dysplasia.

### Study site, period & sample

The GCEP consists of a decentralized service network involving the National University Hospital (NUH), Tan Tock Seng Hospital, Singapore General Hospital and Changi General Hospital in Singapore. This study used data from the NUH only, as it is a tertiary medical institution as well as the programme initiator. The GCEP can be divided into three distinctive phases: Start-up, Full-Implementation and Closeout [22], with each phase encompassing different activities (Additional file 1). Compared to the Start-up and Closeout phases, the Full Implementation phase (01/04/2004 – 31/12/2010) captured most of the cost-intensive activities, and thus closely reflected cost generation assuming the GCEP was officially implemented on a national scale. Therefore this study chose the first 6.5 years from 01/04/2004 to 31/10/2010 of the Full Implementation phase as the costing period. To ensure a minimum 2-year follow-up for every subject, we concentrated our analysis on the cohort recruited between 01/04/2004 and 31/03/2008 (n = 749), from which a random sample of 216 cases (29%) was drawn through proportionate stratified sampling by age, gender and risk profile.

### Resource quantification and costing

A task force funded solely by the GCEP grant was established at the NUH to exclusively operate the programme. The GCEP as the service provider paid the NUH for clinical, logistic and financial services. A co-payment system was also applied for clinical services whereby the GCEP provided free follow-up endoscopy, histology/biopsy and urease testing and patients were liable for baseline endoscopy, medication, consultation and diagnostic tests prescribed at follow-ups (Additional file 2).

From the NUH GCEP database, we retrospectively identified and quantified the resources consumed by a single subject at baseline and subsequent follow-ups by reviewing clinical casenotes and GCEP financial statements. Data sources of cost information included the Land Transportation Authority, Ministry of Manpower and the NUH financial office. The costs of each GCEP service were estimated by multiplying the quantities of various resources with the best-available unit cost for that resource. The cost components and estimation methods are summarized in Table 1.

**Table 1:** Cost components and cost estimation of the GCEP (NUH) (2004–2010)

**Components**	**Items**	**Estimate methods**	**Data source**	**Resource quantification (per GCEP service)**	^*^unit cost
Patient					
Clinical	Medications	Micro-costing	Casenotes & NUH	Name & dosage of medication	Price charged by NUH
	Consultation	Case-mix	Casenotes & NUH	Specialist consultation within 3 months after OGD	Mean charge by NUH
	Diagnostic test	Case-mix	Casenotes & NUH	Tests prescribed at follow-up	Mean charge by NUH
	Histology	Case-mix	Casenotes & NUH	Biopsy during OGD	Mean charge by NUH
	Endoscopy	Case-mix	Casenotes & NUH	Baseline & opportunistic OGD	Mean charge by NUH
Non-clinical	Transportation	National Mean	Casenotes & LTA	Round trip with mean mileage	Taxi fare based on mileage by year
	Patient time	Human capital approach	Casenotes & MOM	One day prescribed	Median age-gender specific wage by year
	Caregiver time	Human capital approach	Casenotes & MOM	One day prescribed	Median gross wage by year
GCEP					
Clinical	Endoscopy	Case-mix	Casenotes & NUH	Endoscopy & related procedure	Fixed charge negotiated with NUH
	Histology	Case-mix	Casenotes & NUH	Biopsy during OGD	Fixed charge negotiated with NUH
	Urease	Case-mix	Casenotes & NUH	Urease test during OGD	Fixed charge negotiated with NUH
	Doctor’s time for OGD	Case-mix	Casenotes & NUH	Mean duration for OGD	Mean salary/minute
Non-clinical	Consumables	Direct allocation	Project record	Total spending/caseload	
	GCEP staff	Direct allocation	Project record	Total salary/caseload	
	Overhead	Direct allocation	Project record	Total spending/caseload	
	Capital	Direct amortization and allocation	Project record	Annual equivalent/caseload	

*NUH*, National University Hospital; *OGD*, oesophago-gastro-duodenoscopy; *LTA*, Land Transportation Authority; *MOM*, Ministry of Manpower.

^*^Nine items used means, two items used medians, four items used direct allocation based on weight and yearly totals.

As this study was conducted in 2010, the unit costs of clinical items were based on actual hospital charges at that time. Although hospital charges may not truly represent the cost of the services provided, these were the only data available. Considering that NUH is a not-for-profit public healthcare institution with most charges being set based on the principle of cost recovery, the use of charges in this instance would be a reasonable reflection of actual costs.

Non-clinical GCEP resources included capital, overhead costs, consumables and manpower, of which only yearly total expenditures were available. Equipment, particularly computers, was the major capital outlay for the GCEP. Therefore the 5-year useful life of a computer was used to calculate the annual equivalent of capital cost [23]. The overhead cost was a 20% increment of cash flow charged every year by the NUH to cover the office space, utilities, logistics and other services. The total amount of the GCEP staff salaries was used to estimate the cost of manpower. Unlike clinical resources, for which the consumption was recorded individually in the casenotes, non-clinical resources were shared by all the subjects served during a given period. Thus the cost of the non-clinical items, the so called ‘programme cost’ were directly allocated to each subject [17,22]. Given that the telephone interview, clinic visit and OGD examination consumed different amounts of time and non-clinical resources, they were assigned as ‘1’, ‘2’ and ‘3’ unit weight respectively to reflect the relative utilization of these resources. The programme cost was then assigned to each subject based on their individual weights. Caregiver time refers to when patients were accompanied by a person other than a nurse to the NUH.

### Outcomes

All individual items were categorized into four components, from which primary outcomes originated for this study (Table 1). These outcomes comprised four cost indices that were informative and essential to future cost-effectiveness analysis and programme budgeting [24]. These indices (expressed as US$ per capita) were: 1) Overall Cost (which includes Patient Clinical, Patient Non-clinical, GCEP Clinical and GCEP Non-clinical) quantifying the overall resource consumption; 2) Clinical Cost (which includes Patient Clinical and GCEP Clinical) quantifying the consumption of clinical resources; 3) GCEP Cost (which includes GCEP Clinical and GCEP Non-clinical) quantifying the economic burden on the health care provider – the GCEP; and 4) Personal Cost (which includes Patient Clinical and Patient Non-clinical) quantifying the cost for a subject to receive GCEP services. These cost indices were presented in 2004 US dollars with 3% discount rate [25].

### Statistical analysis

All four cost indices were computed for each subject in every financial year within the costing period. The Student’s *t*-test, Chi-Square test and survival analysis were used to compare continuous variables, categorical variables and rates between the sample and the cohort respectively. Since economic data are generally right-skewed and right-censored [26], this study used the semi-parametric bootstrapping method (n = 1000) to calculate standard errors. The data in each year of the same subject was correlated across follow-up time. To adjust for this within-subject correlation, the multivariate Generalized Estimation Equation (GEE) was used to model the data and to quantify and test the potential temporal trends in outcome indices. Due to the co-payment system, the temporal trends of the GCEP Cost and Personal Cost would be biased if baseline OGD was included in the analysis. Hence, we excluded the baseline data from the analysis of these two indices. As year 2004 had only baseline OGDs, the data from this year was not presented in the results of the GCEP Cost and Personal Cost. Statistical analyses were performed using SPSS (version 19; SPSS, Inc, Chicago, IL), STATA version 10 (Stata Corporation, TX) and Microsoft Excel 2007 (Microsoft, Redmond, WA). A p-value of 0.05 was used for significance for all statistical analyses.

## Results

The study sample adequately represented the NUH GCEP cohort (Table 2). With the exception of the follow-up time, which was 2.5 months longer for the study sample (p = 0.03), the sample and cohort were homogenous with respect to demographics, patient outcome and event rates with non-significant p values.

**Table 2:** Characteristics of the study cohort and sample

		Cohort^* ^**(n = 749)**	Sample^* ^**(n = 216)**	**P**
Age (year)		60.13 (7.32)	7.32 (60.23)	0.86^*†*^
Age group	50-59 years	426 (56.88)	121 (56.02)	
	≥ 60 years	323 (43.12)	95 (43.98)	0.88^*‡*^
Gender	Male	401 (53.54)	123 (56.94)	
	Female	348 (46.46)	93 (43.06)	0.39^*‡*^
Risk profile	Moderate	211 (28.17)	59 (27.31)	
	High	538 (71.83)	157 (72.69)	0.86^*‡*^
Follow-up (year)		3.34 (1.28)	3.55 (1.22)	0.03 ^*†*^
Outcome	EGC	6 (0.8)	1 (0.46)	
	Death	7 (0.93)	2 (0.93)	
	Drop-out	49 (6.54)	8 (3.70)	
	Survival	687 (91.73)	205 (94.91)	0.47^*‡*^
Incidence Rate^‖^		240 (108, 534)	131 (18, 930)	0.98 ^*§*^
Death Rate^‖^		280 (133, 587)	262 (66, 1048)	0.978 ^*§*^
Drop-out Rate^‖^		1959 (1481, 2592)	1049 (524, 2097)	0.831 ^*§*^

Abbreviation: *EGC*: early gastric cancer.

^*^ Values are the mean (SD) for continuous variables and n (%) for categorical variables. Rates are reported with 95% confidence interval.

^†^ Student *t*-test.

^*‡*^ Chi-Square test.

^*§*^ Fisher’s exact test based on rate difference.

^‖^ Unit of rates is 1/100,000 per year.

**Figure 1:**
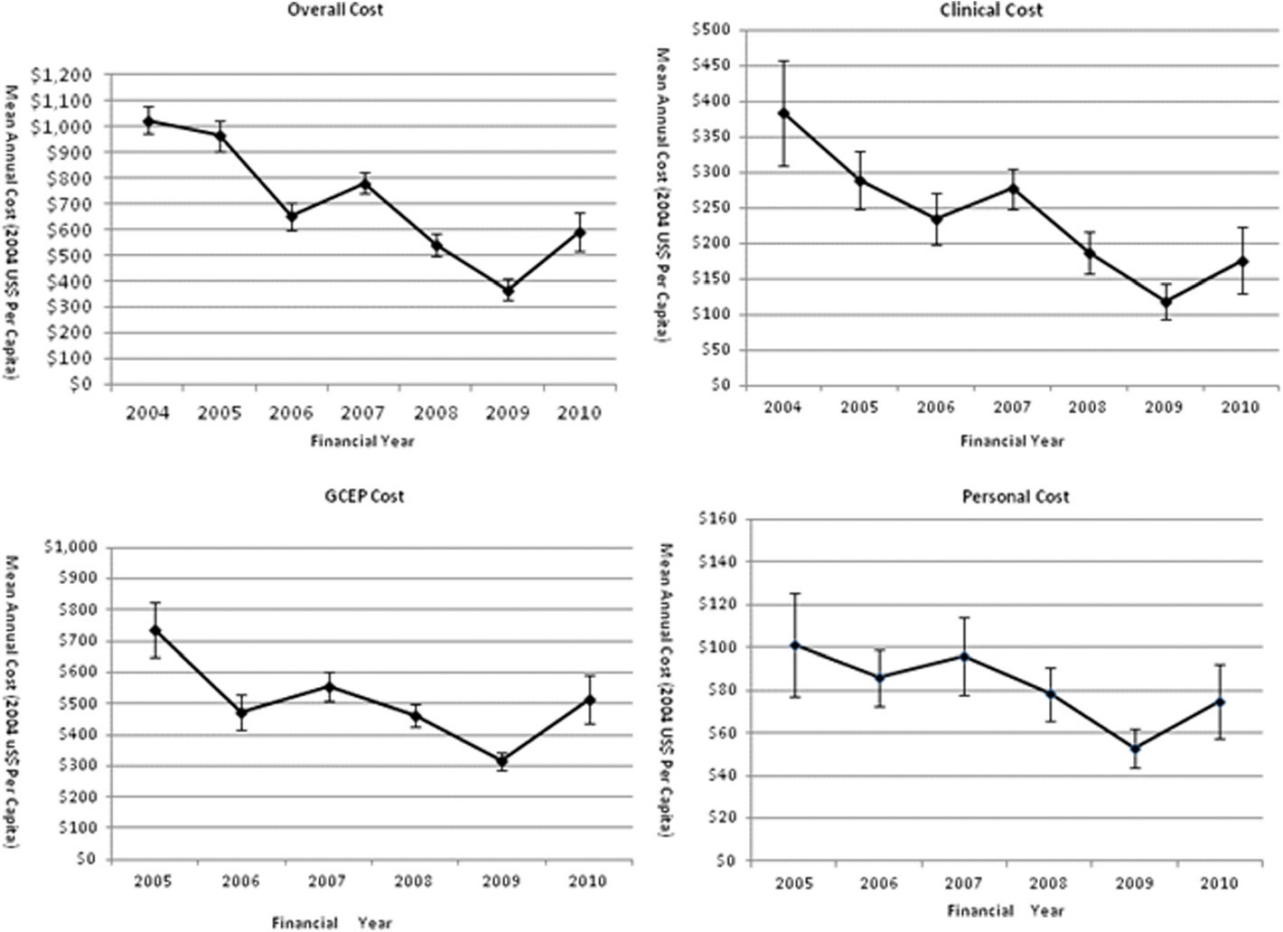
**Temporal trends (2004–2010) of cost indices for the whole sample.** Individual points represent the means of cost indices in each follow-up year.

The cost efficiency of the GCEP improved over time. As shown in Figure 1, the monetary value of all four cost indices declined throughout the 6.5-year costing period. Despite variations over time, the downward trends of the cost indices were apparent from the onset of the GCEP. The mean Overall Cost of serving one subject steadily declined by 42.3% from US$1025 in 2004 to US$591 in 2010. The Clinical Cost, GCEP Cost, and Personal Cost also declined by 54.1%, 30% and 25.7% over this period respectively. The difference in magnitude of cost reduction is the result of the reductions of the cost components constituting each index.

**Figure 2:**
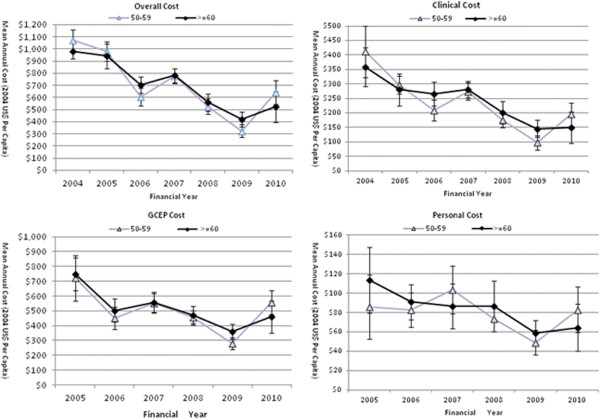
**Temporal trends (2004–2010) of mean cost indices for the age subgroups.** Individual points represent the means of cost indices in each follow-up year.

**Figure 3:**
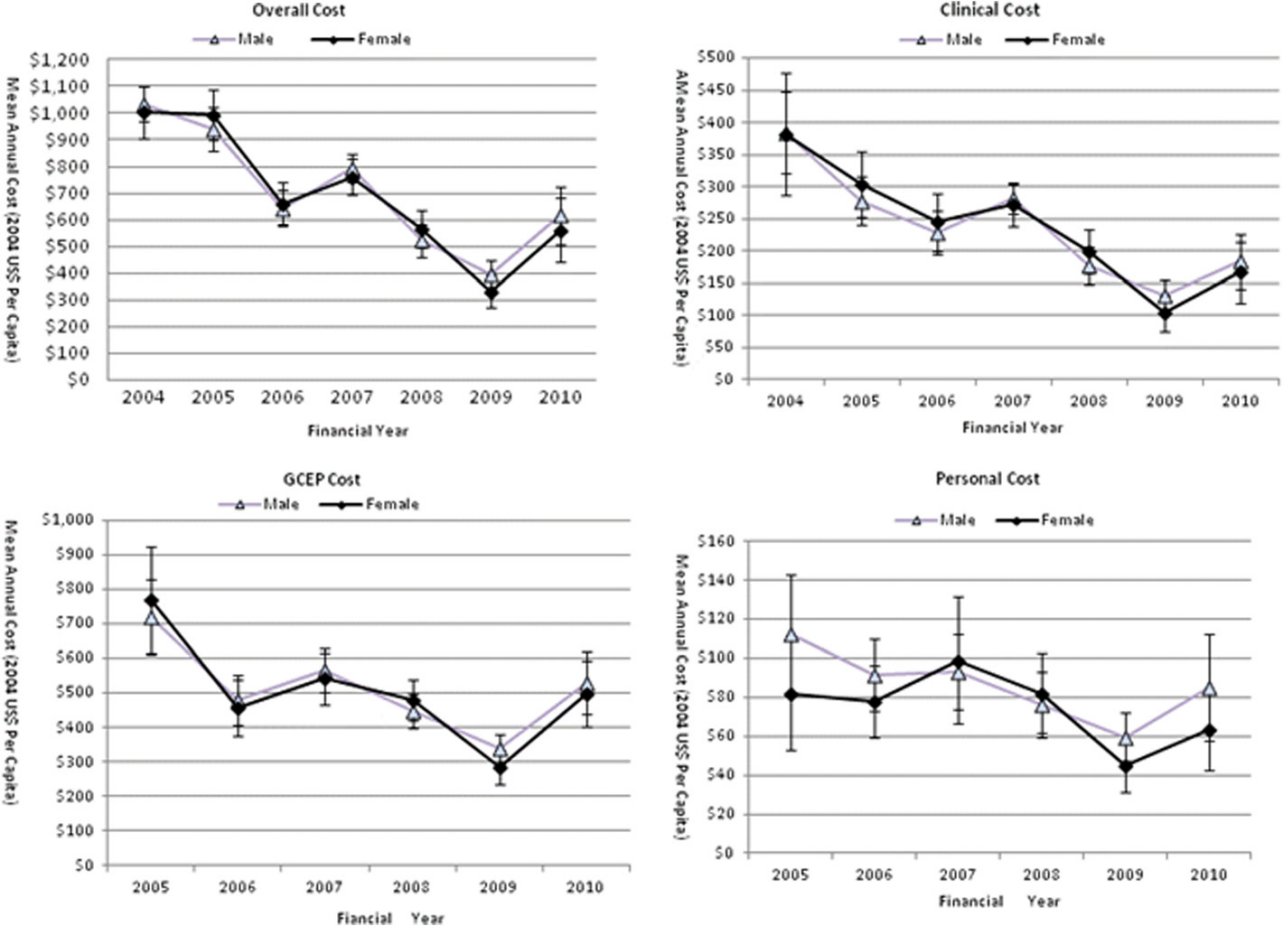
**Temporal trends (2004–2010) of mean cost indices for the gender subgroups.** Individual points represent the means of cost indices in each follow-up year.

As age and gender are critical for defining the target population for GC surveillance, the downward trends of the cost indices were further investigated in the age (50–59 year vs. ≥ 60 year) and gender (male vs. female) subgroups. In Figures 2 and 3, cost indices experienced a slow and steady decline in all four demographic subgroups as they did in the whole sample. Across the four groups, the Overall Cost dropped by between 40% and 47%. The Clinical Cost dropped by 52.1% to 58.4% and the GCEP Cost dropped by between 23% and 39%. The Personal Cost had the least percentage drop of 3.5% for the age group 50–59 years, and the biggest drop of 43% for the age group ≥ 60 years. The downward trends were demonstrated to be consistent for age and gender subgroups. Furthermore, Figures 2 and 3 illustrated that the curves representing subgroups based on age or gender overlapped to a large extent and were almost identical, suggesting that resource consumption was not associated with a subject’s age or gender.

**Figure 4:**
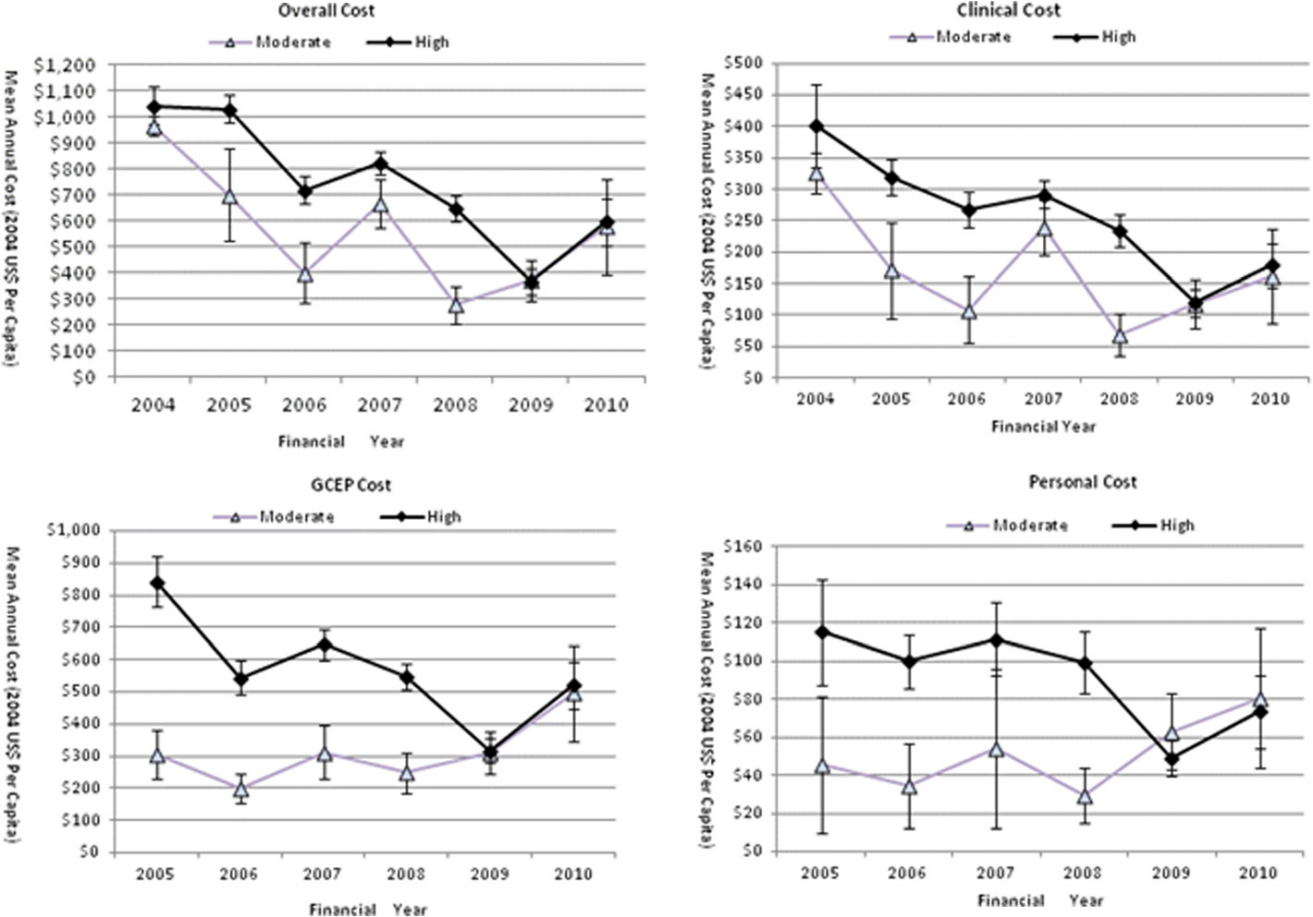
**Temporal trends (2004–2010) of cost indices for the risk subgroups.** Individual points represent the means of cost indices in each follow-up year.

As per protocol, risk assessment at baseline determines the number of OGD which a GCEP subject will take during follow-up and is an important modifiable factor for resource allocation. As expected, the risk profile had a big impact on cost indices for the two risk groups. In Figure 4, the cost curves for the high risk group ran above those of the moderate risk group for all cost indices, illustrating that the high risk subjects consumed more resources than the moderate risk subjects. The Overall Cost, Clinical Cost, GCEP Cost and Personal Cost of the moderate risk group accounted for 77%, 68.1%, 60% and 65.1% of the high risk group respectively.

Risk profile also affected the downward trend pattern. The temporal trends were not universally downward for the three pairs of risk groups or for all cost indices (Figure 4). The Overall Cost and Clinical Cost retained their downward trends for both the high risk and moderate risk groups. However, the GCEP Cost and Personal Cost differed dependent on the risk profile. On average, the GCEP Cost to serve one subject at the high risk dropped by 38.2%, while it increased by 62.6% for a moderate risk subject over the costing period. Similarly, the Personal Cost paid by a single high risk subject dropped by 36.5%, while it increased by 76.1% from US$46 in 2005 to US$81 in 2010 in moderate risk subjects.

The GEE models fitting the temporal trends of cost indices and the comparison of the trends between the three pairs of subgroups of age, gender and risk profile are presented in Table 3. Twenty six out of 28 GEE models in Table 3A confirmed the downward trends as shown in Figures 1, 2, 3 and 4, with highly significant p values and negative annual change in monetary values. Only two GEE models fitting the GCEP Cost and Personal Cost for the moderate risk group suggested a cost increase over the follow-up period. Comparative analysis of the cost trends in the subgroups revealed how other factors affected resource allocation. Significant results were only found when more resources were allocated to the high risk group than to the moderate risk group (Table 3B). There were no significant differences in annual expenses within age or gender subgroups.

**Table 3:** GEE models fitting temporal trends of cost indices and comparison of subgroup trends

		**Overall cost**			**Clinical cost**			**GCEP cost**			**Personal cost**		
		**Mean**	**95% CI**	**P**	**Mean**	**95% CI**	**P**	**Mean**	**95% CI**^**‡**^	**P**	**Mean**	**95% CI**^**‡**^	**P**
**A: Temporal trends**													
Whole Sample^*^		−106	(−117, -95)	< 0.001	−40	(−46, -34)	< 0.001	−48	(−63, -34)	< 0.001	−9	(−12, -4)	< 0.001
Age (year) ^*^	50-59	−109	(−124, -94)	< 0.001	−41	(−49, -33)	< 0.001	−40	(−60, -19)	< 0.001	−7	(−12, -1)	0.01
	> = 60	−102	(−117, -87)	< 0.001	−37	(−47, -28)	< 0.001	−59	(−78, -39)	< 0.001	−10	(−15, -5)	< 0.001
Gender^*^	Male	−101	(−115, -87)	< 0.001	−37	(−45, -29)	< 0.001	−44	(−62, -26)	< 0.001	−7	(−12, -1)	0.009
	Female	−112	(−131, -93)	< 0.001	−43	(−53, -34)	< 0.001	−54	(−77, -32)	< 0.001	−10	(−15, -4)	0.001
Risk Profile^*^	High	−112	(−123, -100)	< 0.001	−43	(−49, -36)	< 0.001	−73	(−87, -60)	< 0.001	−13	(−17, -10)	< 0.001
	Moderate	−46	(−61, -32)	< 0.001	−18	(−28, -7)	0.001	26	(3, 49)	0.027	5	(−3, 14)	0.212
**B: Comparisons between subgroups**													
> = 60 year vs. 50-59^†^		21	(−23, 65)	0.36	17	(−5, 40)	0.127	34	(−17, 84)	0.188	4	(−11, 18)	0.634
Male vs. Female^†^		10	(−35, 54)	0.675	1	(−21, 22)	0.965	15	(−34, 65)	0.536	7	(−8, 21)	0.382
High vs. Moderate^†^		192	(155, 228)	< 0.001	87	(68, 104)	< 0.001	226	(190, 263)	< 0.001	39	(26, 52)	< 0.001

Abbreviations: *C.I*., confidence interval.

^*^ Means are average decrement/increment across costing period.

^†^ Means are differences between two subgroups across costing period.

## Discussion

To the best of our knowledge, studies about continuous cost generation of cancer prevention programmes have not been previously reported despite that many studies have acknowledged the limitations of cross-sectional cost estimates. Our study attempts to fill this gap. The gradual and continuous decline of cost indices in our study strongly indicated the ever-improving cost efficiency of endoscopic surveillance for GC through the GCEP during the observation period. This study as a free standing cost analysis, despite its inability to compute the cost-effectiveness ratio, provided important empirical evidence for programme management and ultimately for value-for-money decision making. Cost studies of long-term GC surveillance have long been anticipated worldwide given that research already illustrated the benefit of GC surveillance [27,28]. Exploring the mechanisms underlying our results would be of universal interest to GC researchers.

Economy of scale is considered the main reason for the cost reduction, especially for the Overall Cost and the GCEP Cost [16,17]. Previous studies have shown that as the screening volume increased, the average cost borne by the health care provider to serve one subject decreased, thereby approximating an inverse relationship [29]. The GCEP has experienced a 7-fold increase of patient volume from 175 in 2004 to 1223 in 2009. Consequently the average cost decreased because fixed costs spread out horizontally across the number of subjects served every year and vertically along the implementation time. Correlation analysis showed a negative correlation coefficient between workload and Overall Cost and GCEP Cost, with the former achieving statistical significance (r = −0.821, p = 0.023).

More efficient utilization of the resources within the GCEP system was another factor driving the GCEP Cost down. It is well known that public health programmes can improve operational efficiency through self-learning [15], which would in return lead to decreased costs borne by the service provider. Having been in operation for seven years and with quality assurance protocols in place, the GCEP could optimize work-flow processes by shortening waiting times, avoiding repetitions and enhancing service awareness in team members [30]. Although specific parameters were not set to gauge its work-flow processes, it was fair to assume that the self-learning mechanism took effect, especially in the inception of the Full Implementation phase which was the costing period of our study.

The decline in Clinical Cost indicated that subjects consumed less of the clinical services, which consist of follow-up OGD, specialist consultations, diagnostic tests and medications (Table 1). This is most likely due to a reduced demand of clinical services in later follow-ups when the subjects were experiencing fewer symptoms as a result of surveillance and associated treatment. Our findings were consistent with a cost study of a colorectal cancer screening programme which showed that repeated screenings cost less than initial screenings because of the lower prevalence of disease in the rescreening group as opposed to first-time participants [31]. Congruent with the previous observation, the Personal Cost also decreased as patients paid less for clinical services.

A further reason for the Personal Cost reduction was the declining price of patient time estimated by the human capital approach [32], whereby the opportunity cost of taking one day off work for an OGD or clinic visit was measured as a single day’s salary. In Singapore, there was a large decrease in salary from the age group 50–59 years to the age group 60 years or above [33]. As one of the GCEP inclusion criteria was being age 50 years old or above, we noted that during the observation period, 42 subjects (19.4%) underwent the age change from 50–59 years old to 60 years old and above.

Subgroup analysis was conducted to explore the cost generation in subgroups categorized by age and gender, which are relevant to the diagnostic yield of a screening programme [34,35]. Similar to the observation for the whole sample, both gender subgroups experienced significant annual decreases with bigger decrements in females for all four cost indices (Table 3). As for the impact of age, compared with subjects 60 years or older, subjects between 50 and 59 years had a larger decrement in Overall Cost and Clinical Cost, US$109 vs. US$102 and US$41 vs. US$37 respectively, and a smaller decrement in the GCEP Cost and Personal Cost, US$40 vs. US$59 and US$7 vs. US$10 respectively (Table 3). Comparing the average costs of the subgroups for either variable failed to reveal significant differences, as illustrated by the overlapping curves in Figures 2 and 3. The cost efficiency in subgroups as described above was of great significance in advising resource distribution among these subgroups and in computing population specific cost-effectiveness ratios subsequently.

As the cost-effectiveness of screening is sensitive to disease incidence in target populations [11,36], the GCEP classified subjects into high and moderate risk of GC, which subsequently determined the frequency of surveillance OGD. The temporal trends of cost indices were statistically different between high and moderate risk groups (Figure 4 and Table 3). The Overall Cost and the Clinical Cost for the high risk group had annual decrements 2.4 times lower than those for the moderate risk group. The GCEP Cost and Personal Cost showed a downward trend in the high risk group, while they both increased over time in the moderate group. The cost difference between high and moderate risk groups was arbitrary as OGD frequencies were decided beforehand, yet it has implications for funding and for evaluating the cost-effectiveness of a specific population.

Compared to other published cost-analyses of cancer screening programmes, our study was unique in four ways, in that we 1) analyzed long-term continuous cost generation; 2) collected individual-level data; 3) identified and quantified all possible resources; and 4) studied multiple indices simultaneously. The advantages of these are discussed as follows.

Given that long-term or life-long follow-up is required in a cancer surveillance programme we studied a prospective cohort, the GCEP, with 6.5-year follow-up data and reported on the temporal trends of cost indices, in addition to the point estimates which are the sole outcomes in cross-sectional studies [13,17]. There is a high likelihood that point estimates are skewed or biased depending on the period chosen in a specific study [14,37]. Programme activities and patient volume varied greatly from year to year resulting in inflated/deflated point estimates [13,38]. Our study, rather than overestimating/underestimating the cost values, reported on the temporal variation of costs that can be used to predict the variability and the evolution of the cost - two aspects crucial for programme budgeting.

Regarding the quality of data, our study collected individual-level data based on the NUH GCEP database. The quality of our data afforded statistical advantages over aggregate data analyzed in other studies [13,39]. Individual-level data captured person-to-person and year-to-year variations which allowed us to estimate the means and confidence intervals from actual distributions and to apply GEE models, thereby enhancing the validity and reliability of our results.

In addition, we used the original documents of patient casenotes and the GCEP financial statements to identify clinical and non-clinical items directly associated with programme operation. A bottom-up approach was adopted to quantify resources consumed and to estimate their monetary value [22,40], thereby avoiding subjectivity or recall bias when data is collected through a survey-based top-down approach [15], and ensuing high accuracy and completeness of the data.

A major contribution of our study was that we simultaneously investigated multiple cost indices, each of which has been a focus in separate previous studies [13,16,32,41]. To our knowledge, no study has investigated these indices simultaneously in a single study thereby overlooking the fact that these costs accrued concurrently. Complete and accurate cost data are crucial to both an economic evaluation and programme planning. Economic evaluations tend to underestimate the cost because of poor representation of personal costs and programme costs [42]. The personal cost represents the financial commitment of a subject to participate in screening [20,41], so it was associated with subject compliance and programme effectiveness [43,44]. Our study found that patients paid at the most 18.2% of what was borne by the service provider (the GCEP) (Figure 1), suggesting that the co-payment could be a viable arrangement. Programme cost measures the expenditure on non-clinical activities and represent the internal resource allocation within programmes. A cost analysis of a colon cancer screening programme demonstrated that non-clinical activities consumed more than 50% of the total budget [17], exceeding the US federal standard of 40% [45]. In our study, the Clinical Cost accounted for only 17.35% to 35.76% of the Overall Cost, i.e., the non-clinical cost ranged between 64.24% and 82.65% (Figure 1). Although this study took a societal perspective and applied a narrower definition of clinical service, as an organized surveillance programme in a small country such as Singapore, a high proportion of non-clinical expenditure appealed to the more efficient internal resource allocation.

We acknowledge several limitations with our study. As a pure cost analysis, this study is inherently unable to inform the value-for-money decision which is of utmost importance yet requires a full economic evaluation. In addition, service underutilization which is negatively associated with programme effectiveness, cannot be ruled out as a mechanism driving down the cost in our study. The co-payment system whereby patients are committed to a certain amount of money could impede some patients from using GCEP services, especially those from low-income families [18,44,46]. Removal of patient costs has been demonstrated to increase the screening compliance [47]. Retrospective data collection in the current study cannot accurately match the cost with the specific clinical or administrative activities. Therefore, we could not identify the area of inefficiency. As for the Personal Cost, we may have omitted some elements which could only be retrieved through personal interview. Furthermore, caution is needed to extrapolate the downward trends beyond the observation period, because all the factors accounting for the cost reduction have limits [17]. Nonetheless, our results confirmed continuous cost decrements in the early phase after full implementation. However, data seemed to indicate that the descending momentum has stopped in 2009 (Figures 1, 2, 3 and 4). The ideal situation is that a programme achieves its optimal cost efficiency and functions on its minimum average cost curve [16]. A measure of a successful programme is how soon this point is reached, however this was not captured in our study.

## Conclusion

Our study highlighted the importance of assessing the cost efficiency of a pilot project for future economic evaluation and government planning. The downward trends in cost indices and the factors contributing towards them offered valuable insights for future programme budgeting and policy making. It is crucial for health administrators and planners to identify these factors and to further maximize their effect on cost efficiency in order for their programmes to succeed. Furthermore, our study illustrated the distinct pattern of resource consumption and its temporal variation in individual subgroups classified by variables defining the target population. These findings call for accurate classification of the target population and for the computation of a population specific cost-effectiveness ratio.

## Abbreviations

GC: Gastric cancer; GCEP: Gastric cancer epidemiology clinical and genetic programme; OGD: Oesophago-gastro-duodenoscopy; NUH: National university hospital; GEE: Generalized estimation equation; LTA: Land transportation authority; MOM: Ministry of Manpower.

## Competing interests

The authors declare that they have no competing interests.

## Authors’ contributions

HJZ designed the study, collected, verified and analyzed the data, drafted and revised the manuscript. SCL critically revised the manuscript, improved the quality of data interpretation and results report, and instructed the discussion. NN critically revised the manuscript, refined the study report, and provided ideas for discussion. FZ coordinated the project and revised manuscript. KGY is the principal investigator. He initiated, funded and approved the study, supervised and monitored the study process and critically revised the manuscript. All authors read and approved the final manuscript.

## Pre-publication history

The pre-publication history for this paper can be accessed here:

http://www.biomedcentral.com/1472-6963/13/139/prepub

## Supplementary Material

Additional file 1 **Phases and time frame of the GCEP.** (TIFF 46 kb) Click here for file

Additional file 2 **Cost structure of the GCEP.** (TIFF 44 kb) Click here for file
