# Supplementary figures and images for: Empirical evidence of the continuing improvement in cost efficiency of an endoscopic surveillance programme for gastric cancer in Singapore from 2004 to 2010
Source: BMC Health Serv Res. 2013 Apr 15;13:139. doi: 10.1186/1472-6963-13-139 (PMC3637081; doi:10.1186/1472-6963-13-139)

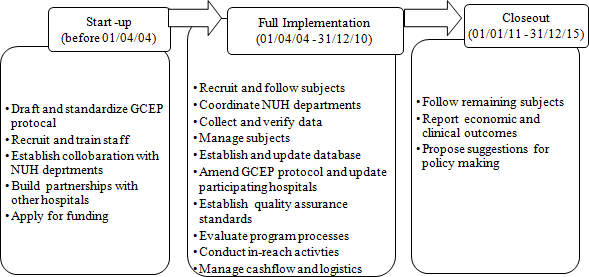

Supplement: Additional file 1 — Phases and time frame of the GCEP. (TIFF 46 kb) [file 1472-6963-13-139-S1.tiff]

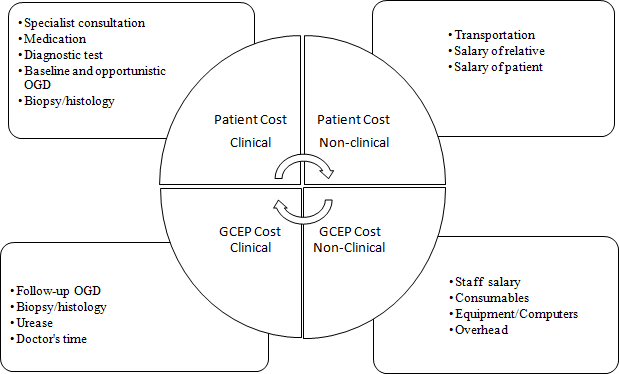

Supplement: Additional file 2 — Cost structure of the GCEP. (TIFF 44 kb) [file 1472-6963-13-139-S2.tiff]
